# Genome-wide characterization and evolutionary analysis of linker histones in castor bean (*Ricinus communis*)

**DOI:** 10.3389/fpls.2022.1014418

**Published:** 2022-10-21

**Authors:** Jiayu Guo, Ping Li, Anmin Yu, Mark A. Chapman, Aizhong Liu

**Affiliations:** ^1^ Key Laboratory for Forest Resource Conservation and Utilization in the Southwest Mountains of China, Ministry of Education, Southwest Forestry University, Kunming, China; ^2^ Biological Sciences and Centre for Underutilised Crops, University of Southampton, Southampton, United Kingdom

**Keywords:** castor bean, linker histone, globular domain, gene expression, intraspecific variation, genetic diversity, phylogenetic analyses

## Abstract

H1s, or linker histones, are ubiquitous proteins in eukaryotic cells, consisting of a globular GH1 domain flanked by two unstructured tails. Whilst it is known that numerous non-allelic variants exist within the same species, the degree of interspecific and intraspecific variation and divergence of linker histones remain unknown. The conserved basic binding sites in GH1 and evenly distributed strong positive charges on the C-terminal domain (CTD) are key structural characters for linker histones to bind chromatin. Based on these features, we identified five linker histones from 13 GH1-containing proteins in castor bean (*Ricinus communis*), which were named as RcH1.1, RcH1.2a, RcH1.2b, RcH1.3, and RcH1.4 based on their phylogenetic relationships with the H1s from five other economically important Euphorbiaceae species (*Hevea brasiliensis Jatropha curcas*, *Manihot esculenta Mercurialis annua*, and *Vernicia fordii*) and *Arabidopsis thaliana*. The expression profiles of *RcH1* genes in a variety of tissues and stresses were determined from RNA-seq data. We found three *RcH1* genes (*RcH1.1*, *RcH1.2a*, and *RcH1.3*) were broadly expressed in all tissues, suggesting a conserved role in stabilizing and organizing the nuclear DNA. *RcH1.2a* and *RcH1.4* was preferentially expressed in floral tissues, indicating potential involvement in floral development in castor bean. Lack of non-coding region and no expression detected in any tissue tested suggest that *RcH1.2b* is a pseudogene. *RcH1.3* was salt stress inducible, but not induced by cold, heat and drought in our investigation. Structural comparison confirmed that GH1 domain was highly evolutionarily conserved and revealed that N- and C-terminal domains of linker histones are divergent between variants, but highly conserved between species for a given variant. Although the number of *H1* genes varies between species, the number of H1 variants is relatively conserved in more closely related species (such as within the same family). Through comparison of nucleotide diversity of linker histone genes and oil-related genes, we found similar mutation rate of these two groups of genes. Using Tajima’s *D* and ML-HKA tests, we found *RcH1.1* and *RcH1.3* may be under balancing selection.

## Introduction

H1 histones (linker histones) are ubiquitous components of eukaryotic chromatin and act to stabilize the nucleosome and promote chromatin organization into higher-order structures. The typical linker histones are characterized by a tripartite structure consisting of a highly conserved globular domain (GH1) flanked with two unstructured tails, a short N-terminal domain (NTD) and a longer C-terminal domain (CTD). Instead of recognizing specific DNA sequence sites, linker histones usually function as architectural proteins by recognizing specific DNA geometry (minimally including a solitary nucleosome with a single DNA linker arm) to induce chromatin conformation change through simultaneous and synergistic binding of the GH1 and CTD regions to their targets (Stasevich et al., 2010; White et al., 2016). The GH1 domain, characterizing the winged-helix protein family, consists of a three-helix bundle (helix I, II, III) connected with a C-terminal β-hairpin, and binds to the nucleosome in two states through two major sites that are formed by two clusters of highly conserved basic residues (Goytisolo et al., 1996; Brown et al., 2006; Woods and Wereszczynski, 2020). In the ‘on-dyad’ state, the GH1 domain is centered on the dyad of the nucleosome with site I binding to the nucleosomal core DNA and one linker DNA, and site II interacting with another DNA linker (Zhou et al., 2015; Bednar et al., 2017). In the ‘off-dyad’ configuration, the GH1 domain sits askew from the dyad axis bridging the nucleosomal DNA near the dyad axis and one 10-base pair linker DNA asymmetrically by the two binding sites (Zhou et al., 1998; Zhou et al., 2013). The NTD has little effect on chromatin binding (Syed et al., 2010), however the CTD plays an indispensable role in assisting correct GH1 placement through its abundant and evenly distributed lysine residues to neutralize negative charges on targeted linker DNA (Lu and Hansen, 2004; Stasevich et al., 2010). Therefore, the GH1 domain and CTD are the crucial basis for linker histone functioning, and also provide key characters for the identification of linker histones.

In addition to stabilization of the nucleosome and organization of chromatin structures, linker histones are often involved in regulating embryonic or larval development in animals (Fan et al., 2003; Fan et al., 2005; Lu et al., 2009). In plants, studies in tobacco have revealed the crucial roles of linker histones (such as H1A and H1B) for directing male meiosis, the development of pollen grains, and temporal expression patterns of certain genes regulating floral development (Prymakowska-Bosak et al., 1999; Przewloka et al., 2002). Suppression of H1 genes in *Arabidopsis* resulted in heritable developmental defects and stochastic changes in DNA methylation (Wierzbicki and Jerzmanowski, 2005). In particular, linker histones mediate DNA methylation of euchromatin and heterochromatin, and are involved in regulating genomic epigenetic modifications in *Arabidopsis* (Zemach et al., 2013; Choi et al., 2021). Specific linker histones (such as H1.3) function in the regulation of plant growth in response to abiotic stresses (Ascenzi and Gantt, 1997; Scippa et al., 2000; Przewloka et al., 2002; Rutowicz et al., 2015). Thus, it seems that linker histones are broadly involved in participating the regulation of plant growth and development.

Diverse non-allelic variants of linker histones have been found in both animals and plants, including species-specific, cell type-, stage-specific, and stress inducible isoforms (Jerzmanowski et al., 2000; Izzo et al., 2008; Sancho et al., 2008). Compared to the high conservation of core histones (H2, H3, and H4), linker histones are highly variable and divergent especially among more divergent species (Jerzmanowski et al., 2000; Kotliński et al., 2017), yet little is known about variation feature within a given species or between populations in evolutionary, or how orthologous linker histone variants vary between species.

Castor bean (*Ricinus communis* L., 2n=20) is, an important oilseed crop, and a member of the Euphorbiaceae family, which contains several economically important species. Owing to the high content of ricinoleic acid in its seed oil, castor oils have been widely used in various industries, including engine lubricants, biofuels, cosmetic and coatings (Da Silva et al., 2006; Ogunniyi, 2006). Due to the economic importance and the wide adaptability to diverse soil and climate conditions, castor bean has been widely introduced and cultivated in many countries and regions all over the world. The annual castor cultivars were domesticated from wild tree-like germplasm distributed in Kenya and Ethiopia; our previous study has revealed distinct differentiation between the wild and domesticated lines on both the genomic and phenotypic levels (Xu et al., 2021). Data from this study represents an opportunity to investigate the variation of non-allelic linker histones and compare the variation in allelic and non-allelic *H1* genes between wild and domesticated castor bean.

The H1 linker histones of *Arabidopsis* were named based on the phylogenetic relationships among variants by Talbert et al. (2012). In this study, we identified and characterized the linker histones in castor bean and compared these to several important members of Euphorbiaceae, including rubber tree, jatropha, tung tree, cassava, and annual mercury based on genome sequences. We extended the phylogenetical analysis and nomenclature of linker histones for castor bean and other five members in Euphorbiaceae. We investigated the expression of linker histones among different tissues and in response to stresses in castor bean. Further, we analyzed genetic variation and divergence of linker histone genes between wild and domesticated castor bean. This study demonstrates for the first time how linker histones vary within and between populations and species and provides new insight into the potential functions of linker histones within castor bean and potentially more broadly.

## Results

### Identification and structural features of H1s in castor bean

**Figure 1:**
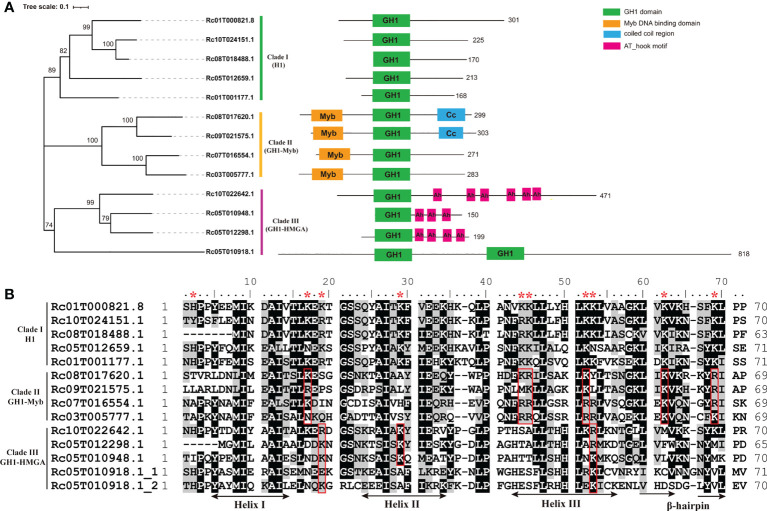
Structural features of GH1-containing proteins in castor bean. **(A)** Neighbor-joining phylogenetic tree and domain architecture GH1-containing proteins in castor bean. **(B)** Amino acid sequence comparison of GH1 domains (Rc05T010918.1 has two GH1 domains). The sites with asterisks above are the putative conserved basic binding sites in clade (I) The sites in the red boxes are the putative conserved basic binding sites residues in clade II or III.

In total, 13 distinct proteins with typical globular domains (GH1) were identified based on the wild castor bean reference genome (Xu et al., 2021) (**Table 1**, **Supplementary Table S1**). One member (Rc05T010918.1) that contains two GH1 domains, and the other 12 members all have a single GH1 domain. When analyzing the domain structures, the 13 proteins formed three major groups. Five members (Rc01T000821.8, Rc10T024151.1, Rc08T018488.1, Rc05T012659.1, Rc01T001177.1) were characterized by one GH1 domain flanked with two unstructured tails, which was consistent with the typical structure of linker histones, with an exception of Rc08T018488.1 that lacked the NTD (**Figure 1A**). Four members (Rc08T017620.1, Rc09T021575.1, Rc07T016554.1, Rc03T005777.1) had an additional Myb domain in the NTD, whilst three (Rc10T022642.1, Rc05T012298.1, Rc05T010948.1) harbored multiple (three to six) AT-hook motifs in the CTD in addition to the GH1 domain (**Figure 1A**). Rc05T010918.1 harbored two GH1 domains, with no other domains (**Figure 1A**).

**Table 1:** Information of the GH1-containing proteins in castor bean.

Protein_ID	MW (kDa)	Theoretical pI	Protein Length (aa)				Lysine content (%)				Net charge			
			total	NTD	GH1	CTD	total	NTD	GH1	CTD	Total	NTD	GH1	CTD
Rc01T000821.8	31.32	10.70	301	64	70	167	23.9	15.6	20.0	28.7	64	3	8	53
Rc10T024151.1	23.51	10.96	225	50	70	105	24.4	22.0	17.1	30.5	53	9	9	35
Rc08T018488.1	18.31	11.16	170	0	63	107	24.7	—	17.5	29.0	43	—	9	34
Rc05T012659.1	22.93	10.3	213	50	71	92	23.5	24.0	15.5	29.3	37	2	8	27
Rc01T001177.1	18.87	10.44	168	20	71	77	23.2	20.0	18.3	28.6	32	4	8	20
Rc08T017620.1	33.23	9.55	299	120	69	110	9.7	10.8	13.0	6.4	10	6	7	-3
Rc09T021575.1	32.91	9.77	303	118	69	116	11.9	13.6	11.6	10.3	16	9	6	1
Rc07T016554.1	29.77	9.01	271	109	69	93	8.5	11.0	5.8	7.5	5	3	6	-4
Rc03T005777.1	31.30	9.08	283	120	69	94	9.9	11.7	8.7	8.5	6	2	7	-3
Rc10T022642.1	48.71	11.03	471	67	70	334	7.2	0.0	11.4	7.8	41	-2	9	34
Rc05T012298.1	16.12	10.43	150	0	65	85	8.7	—	9.2	8.2	13	—	0	13
Rc05T010948.1	21.18	10.96	199	26	70	103	9.0	0.0	10.0	10.7	21	-3	1	23
Rc05T010918.1	93.64	9.42	818	161	71/70	383	10.3	9.9	8.5/10.0	8.9	36	6	3/-1	12

The total lysine content and net charges in CTD of proteins in clade I are in bold.

A neighbor-joining phylogenetic analysis based on amino acid sequence of the whole protein of the 13 members was conducted. As shown in **Figure 1A**, the 13 members split into distinct three clades, consistent with the domain structures. Clade I was comprised of the five members with the typical domain structures of H1s (Rc01T000821.8, Rc10T024151.1, Rc08T018488.1, Rc05T012659.1, Rc01T001177.1), Clade II consisted of the four members with an additional N-terminal Myb domain (Rc08T017620.1, Rc09T021575.1, Rc07T016554.1, Rc03T005777.1), and Clade III comprised the three members with multiple AT-hook motifs (Rc10T022642.1, Rc05T012298.1, Rc05T010948.1) plus Rc05T010918.1 (**Figure 1A**). Although Rc05T010918.1 has no AT-hook motifs, other sequence features place this in the group with the three members with AT-hook motifs.

Since the GH1 domain in linker histone functions to bind DNA, we inspected the structure features of identified GH1 domains, which ranged from 63 to 71 amino acid residues in length (**Table 1**). The typical three helixes (Helix I, II and III) and the β-hairpin of GH1 domain were annotated according to the three-dimensional structure of globular domain of histone H5 of *Gallus gallus* (homologous to H1 and the predominant linker histone in avian erythrocytes) by model building (Ramakrishnan et al., 1993; Goytisolo et al., 1996). In animals, the putative DNA binding sites on the surface of GH1 or GH5 domain have been inferred in many studies (Ramakrishnan et al., 1993; Goytisolo et al., 1996; Brown et al., 2006), which were conserved basic residues including His25 (5’ of Helix I), Lys40 and Arg42 (between Helix I and II), Arg47 and Lys52 (in Helix II), Lys69 and Lys73 (in Helix III), Lys85 and Arg94 (β-hairpin), and Lys97 (3’ to the β-hairpin). According to the position of these binding sites in GH1 domain in animals, conserved basic residues at the corresponding position in GH1 domains of the 13 members in castor bean were investigated. As shown in **Figure 1B**, 10 conserved basic residues at the corresponding positions were observed in the five members in clade I (Rc01T000821.8, Rc10T024151.1, Rc08T018488.1, Rc05T012659.1, Rc01T001177.1), locating 5’ to Helix I (His2), between Helix I and II (Lys17 and Arg/Lys19), in Helix II (Lys29), in Helix III (Lys43, Lys/Arg44, Lys51, Lys52), and in the β-hairpin (Lys61, Lys67). The 3D models of the GH1 domain demonstrated that these 10 residues are all located on or near the surface of the GH1 domain (**Supplementary Figure 1**), therefore they were considered putative binding sites of the GH1 domain in the five members of clade I. Fewer conserved basic residues at the corresponding positions were observed in members of clade II and III. Six of these 10 putative sites were conserved in the four members with Myb domain (clade II), whilst only three were conserved in the four members in clade III (**Figure 1B**).

When inspecting the sequence features of the NTD and CTD of the 13 members, we found high variation in peptide length (**Table 1**). The C-terminal tail (CTD) of linker histone is typically lysine-rich and plays essential roles in assisting GH1 domain binding to DNA by its evenly distributed strong positive charges (Lu and Hansen, 2004; Stasevich et al., 2010). The five members in clade I all showed high lysine content (ca. 30%) in the CTD, which was at least ca. three times higher than that in the members in the other two clades (**Table 1**). We found that the CTD of these five members harbored high positive net charges ranging from 20 to 53 (**Table 1**), and only these five members possessed strong positive charges along the whole C-terminal tail (**Supplementary Figure 2**). In comparison, the CTD of the other eight members (clades II and III) were either negatively or weakly charged, or exhibited intermittent positively charge islands (**Table 1**, **Supplementary Figure 2**).

Based on the evidence above, we consider the five members (Rc01T000821.8, Rc10T024151.1, Rc08T018488.1, Rc05T012659.1, Rc01T001177.1) in clade I to be true linker histone H1s in castor bean. The members of clade II and III were named as GH1-Myb and GH1-HMGA proteins, respectively, according to the nomenclature of orthologues in *Arabidopsis thaliana* (Kotliński et al., 2017). The distinct separation of these three clades and the divergent features among them suggests ancient differentiation between H1s, GH1-Myb and GH1-HMGA proteins in the evolution, with applicability to future phylogeny-based identification of linker histones.

### Phylogeny based nomenclature of H1s and their interspecific variation

In addition to castor bean, we identified the GH1-containing proteins in five related species in the Euphorbiaceae family, including *Hevea brasiliensis*, *Jatropha curcas*, *Manihot esculenta*, *Mercurialis annua* and *Vernicia fordii* (**Supplementary Table S2**). With the addition of castor bean and *Arabidopsis thaliana*, a total of 128 GH1-containing proteins were used to construct a maximum likelihood phylogenetic tree based on the amino acid sequence of the whole protein. As expected, the ML tree supported an early separation into three clades, namely H1 (Clade I), GH1-HMGA (Clade II), and GH1-Myb proteins (Clade III) (**Supplementary Figure 3**), with the exception of one member from *Mercurialis annua* (g34113.t1) which was isolated and sister to the H1 and GH1-HMGA clades, however with poor bootstrap support. The GH1 domain of g34113.t1 suggests it is a GH1-HMGA protein, but again is found as sister to all the other GH1-HMGA proteins (**Supplementary Figure 4**). Further, g34113.t1 lacks the C-terminal tail (**Supplementary Table S2**), therefore we presume it is non-functional. Based on phylogenetic analysis, 38 H1 linker histones, in total, were identified and characterized in castor bean and the other five species in Euphorbiaceae (**Supplementary Figures 3, 4**).

**Figure 2:**
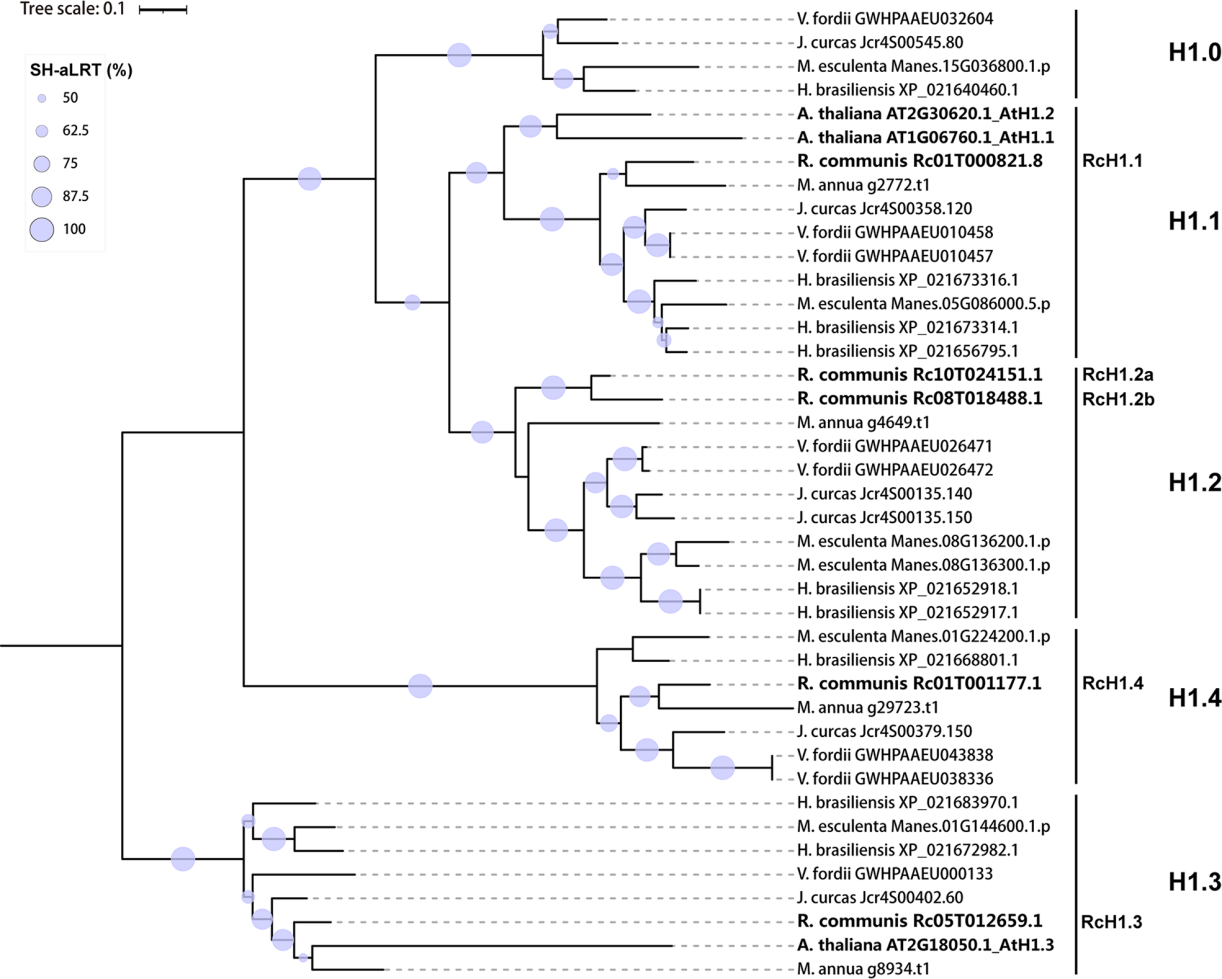
Maximum likelihood phylogenetic tree based on amino acid sequences of H1 linker histones in *Ricinus communis*, *Hevea brasiliensis*, *Jatropha curcas*, *Manihot esculenta*, *Mercurialis annua*, *Vernicia fordii* and *Arabidopsis thaliana*. The branches labeled by light grey circles indicate > 50% SH-like supports of approximate likelihood ratio test (SH-aLRT) and over 50% ultrafast bootstrap support.

To investigate the phylogeny of these identified H1 linker histones for further nomenclature of H1 linker histones we reanalyzed phylogenetical relationships of 38 identified H1 linker histones and the three variants from *Arabidopsis*. As shown in **Figure 2**, the ML tree were nested into five subclades, which were considered five H1 variants and named as H1.0, H1.1, H1.2, H1.3 and H1.4 based on the category and nomenclature in *Arabidopsis thaliana*. The five H1 members from castor bean were found in subclades H1.1, H1.2, H1.3 and H1.4 in the ML tree, and hence were named as RcH1.1, RcH1.2a, RcH1.2b, RcH1.3 and RcH1.4 (**Figure 2**). In terms of the H1 variants in other species, the phylogenetic tree revealed that two H1 variants (H1.1 and H1.3) were present in *Arabidopsis thaliana*, four H1 variants (H1.1, H1.2, H1.3, H1.4) in *Ricinus communis* and *Mercurialis annua*, and all five H1 variants (H1.0, H1.1, H1.2, H1.3, H1.4) in *Hevea brasiliensis Jatropha curcas*, *Manihot esculenta*, and *Vernicia fordii* (**Figure 2**, **Table 2**). The number of H1 variants identified in the six Euphorbiaceae species varied slightly, ranging from 4 to 5, but copy number of each variant varied with species. Species with larger genomes tend to harbor more H1 members (**Table 2** and **Figure 2**).

**Table 2:** Genome size and number of H1 variants and members in the seven species.

Species	Genome size (Mb)	Number of H1 members	Number of copies for each variant					Citation
			H1.0	H1.1	H1.2	H1.3	H1.4	
*Arabidopsis thaliana*	120	3	0	2	0	1	0	(Lamesch et al., 2012)
*Jatropha curcas*	298	6	1	1	2	1	1	(Hirakawa et al., 2012)
*Ricinus communis*	336	5	0	1	2	1	1	(Xu et al., 2021)
*Mercurialis annua*	640	4	0	1	1	1	1	(Veltsos et al., 2019)
*Manihot esculenta*	640	6	1	1	2	1	1	https://phytozome-next.jgi.doe.gov/info/Mesculenta_v8_1
*Vernicia fordii*	1176	8	1	2	2	1	2	(Cui et al., 2018)
*Hevea brasiliensis*	1460	9	1	3	2	2	1	(Tang et al., 2016)

Through sequence comparison, we found that amino acid sequence and length of GH1 domains were highly conserved between different H1 variants and between these seven species (**Supplementary Figure 5**). The 10 putative DNA binding sites in linker histones identified in castor bean were also highly conserved across H1s variants and across species. There were still minor variations between different variants at specific sites, for instance, the putative binding site His2 was replaced by neutral Tyr in H1.0 and H1.2; Lys17 and Lys55 was replaced by neutral Asn in H1.3 (**Supplementary Figure 5**). When inspecting the NTD and CTD, we found high variation in length between variants (as found in the analysis of castor bean H1s above), but relatively high conservation in both sequence and length of the NTD and CTD across species for the same variant (**Supplementary Figures 6, 7**). The lysine content in CTD of H1 linker histones is conserved across variants and across species, with a value of ~30% (**Supplementary Table S3**). These comparisons demonstrated that H1 variants differ in the amino acid sequences and length of NTD and CTD, more so than the GH1 domain.

### Gene expression profile across different tissues and stresses

**Figure 3:**
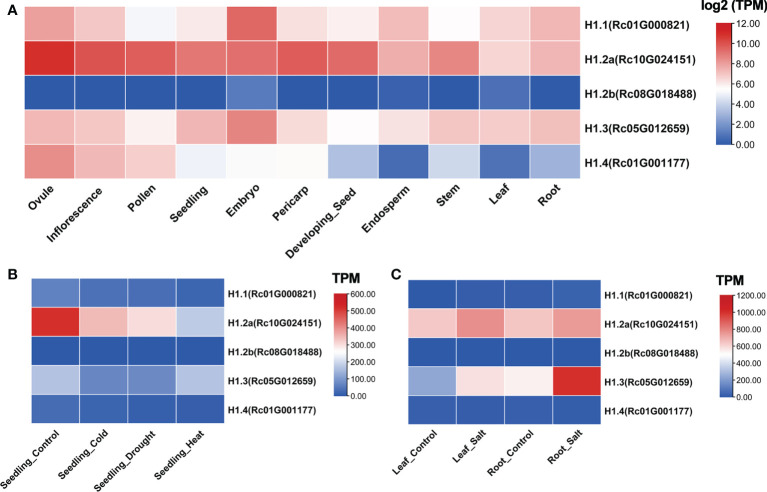
Expression profiles of *H1* genes in castor bean. **(A)** Expression profile across different tissues. Expression is based on the log2(TPM) values. **(B, C)** Expression profile in different stresses. Expression is based on the TPM values.

To inspect the transcriptional features of putative linker histones among tissues in castor bean, we investigated the five *H1* genes using high throughout RNA-seq data obtained from 11 tissues including ovule, inflorescence, pollen, seedling, embryo, pericarp, endosperm, stem, leaf, root, and developing seed (25 days after pollination). These transcription data have been developed and hosted on our website (https://woodyoilplants.iflora.cn/). As shown in **Figure 3A**, *RcH1.1*, *RcH1.2a*, and *RcH1.3* were highly expressed in all tissues tested, whereas *RcH1.2b* expression was hardly detected (TPM ranged from 0-1.2). *RcH1.4* was differentially expressed across the tissues with highest expression in ovules, inflorescences and pollen. *RcH1.2a* also exhibited greater expression in floral tissues than vegetative tissues, whilst *RcH1.1* and *RcH1.3* were most highly expressed in the embryo (**Figure 3A**).

To test whether expression of genes encoding linker histones is sensitive to environmental stresses, we investigated their expression profile under cold, heat, drought, and salt stresses. *RcH1.2a* was down-regulated in seedlings under cold, drought, and heat stresses, whereas expression level of the other genes showed little change across these treatments (**Figure 3B**). Under salt stress, *RcH1.3* was significantly upregulated in both leaves and roots (Fold change > 1.5, *p*-adjust < 0.05; **Figure 3C**), demonstrating that *RcH1.3* was salt-stress inducible. A smaller increase in expression was found under salt stress for *RcH1.2a* in leaves and roots. No obvious changes in expression of the remaining genes were observed under salt stress (**Figure 3C**).

### Population genetic diversity and selection analysis

To investigate variation in *H1* genes at the population level, we utilized the sequences of the five *H1*s from 191 individuals based on our genome resequencing dataset of 106 wild accessions and 85 landraces and cultivars (LC) (Xu et al., 2021). In total, 196 single nucleotide polymorphisms (SNPs) and 40 insertions/deletions (indels) were identified from the five *H1* genes across all individuals, resulting in an average of 1 SNP for every 162.6 bp and 1 indel for every 796.8 bp of sequence (**Supplementary Table S4**). A total of 30 polymorphisms were detected in the coding region in the five *H1* genes with 22 non-synonymous SNPs and 8 synonymous SNPs (**Supplementary Table S4**).

**Figure 4:**
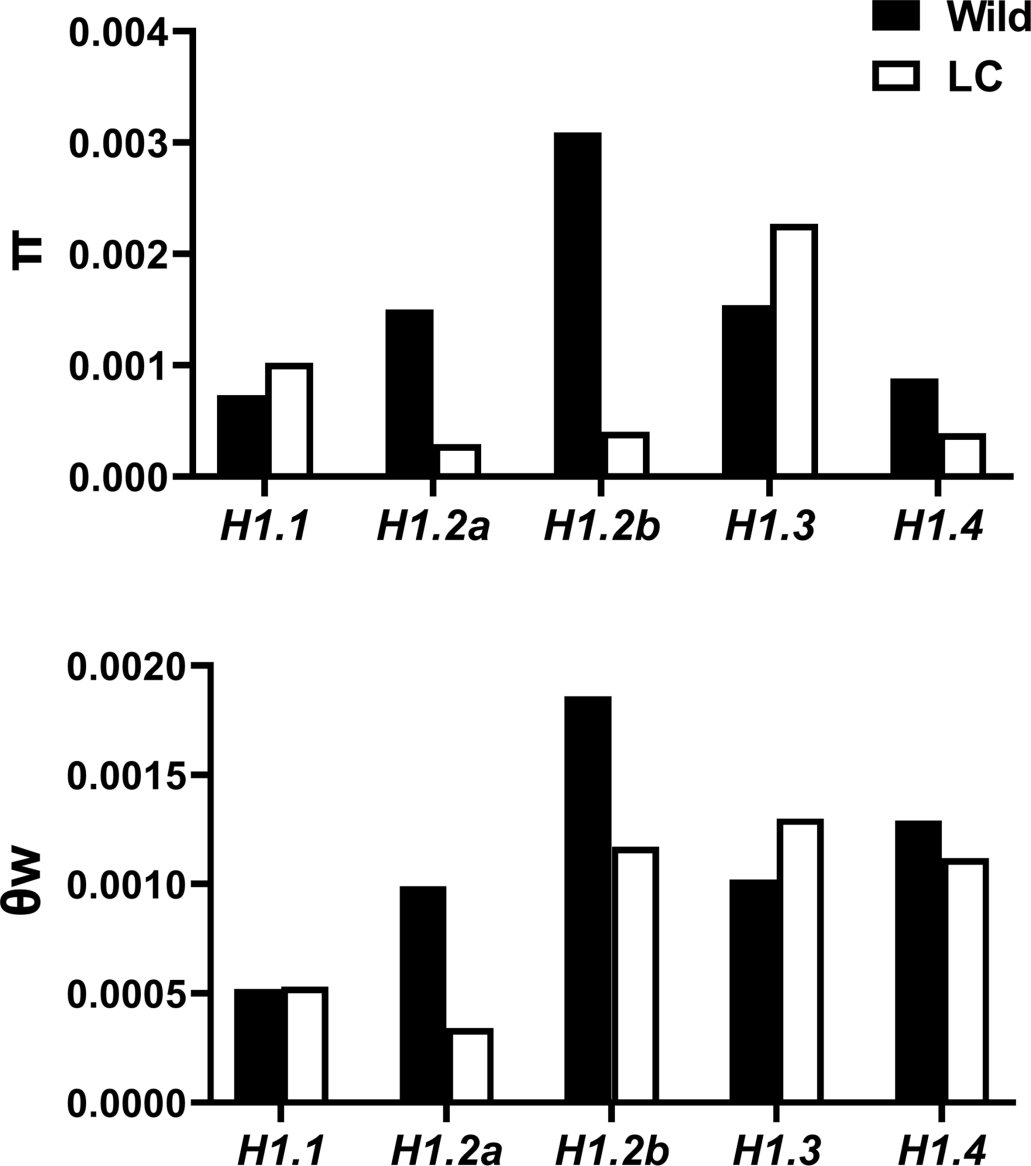
π and θw of *H1* genes in wild and landrace/cultivated (LC) populations.

Nucleotide diversity (π), Watterson’s θ (θw) and Tajima’s *D* were estimated, and Maximum-Likelihood Hudson-Kreitman-Aguade (ML-HKA) tests conducted to infer selection at the five *H1* genes. π and θw for the total population at the five *H1* genes ranged from 0.0007-0.0023 and 0.0006-0.0017 respectively. Generally, genetic diversity in wild population was typically higher than that in LC population (**Figure 4**, **Table 3**).

**Table 3:** Summary of nucleotide diversity, Tajima’s *D* and ML-HKA test at *H1* genes in castor bean.

Gene	Population	θw (total)	π (total)	π (sil)	π (syn)	π (non-syn)	Tajima’s *D*	*p*-value in ML-HKA test
***RcH1.1***	**Total**	**0.0006**	**0.0010**	**0.0009**	**0.0005**	**0.0013**	2.2185*	
	LC	0.0005	0.0010	0.0009	0.0006	0.0014	2.9949**	0**
	Wild	0.0005	0.0007	0.0007	0.0004	0.0009	1.3004	0.00017**
***RcH1.2a***	**Total**	**0.0010**	**0.0011**	**0.0016**	**0.0022**	**0.0004**	0.0447	
	LC	0.0003	0.0003	0.0005	0.0000	0.0000	-0.2697	0.354
	Wild	0.0010	0.0015	0.0022	0.0036	0.0006	1.1649	0.234
***RcH1.2b***	**Total**	**0.0017**	**0.0022**	**0.0000**	**0.0000**	**0.0029**	0.6322	
	LC	0.0012	0.0004	0.0000	0.0000	0.0005	-1.2173	0.421
	Wild	0.0019	0.0031	0.0000	0.0000	0.0041	1.3968	0.595
***RcH1.3***	**Total**	**0.0015**	**0.0023**	**0.0023**	**0.0010**	**0.0014**	1.4798	
	LC	0.0013	0.0023	0.0023	0.0021	0.0010	2.4825*	0**
	Wild	0.0010	0.0015	0.0016	0.0000	0.0008	1.6320	0**
***RcH1.4***	**Total**	**0.0012**	**0.0007**	**0.0007**	**0.0007**	**0.0007**	-0.8215	
	LC	0.0011	0.0004	0.0006	0.0011	0.0002	-1.4341	0.660
	Wild	0.0013	0.0009	0.0008	0.0004	0.0010	-0.7017	0.526

*p<0.05, **p<0.01. Comparisons that resulted significant in Tajima’s D and ML-HKA tests are indicated in bold.

However, these two diversity metrics were quite variable across the five *H1* genes. For the Rc*H1.2a*, *RcH1.2b*, and *RcH1.4*, we found reduced nucleotide diversity (π and θw) in the LC population compared to the wild (**Figure 4**), but no significant evidence of selection was detected using Tajima’s *D* or the ML-HKA test (**Table 3**). For *RcH1.3*, we observed greater nucleotide diversity (π and θw; primarily at the silent sites) in the LC population relative to the wild (**Figure 4**), and this resulted in a significant positive Tajima’s *D* for the LC population and significant ML-HKA tests for both populations (*p*<0.05, **Table 3**). The increased genetic diversity, positive Tajima’s *D*, and significant ML-HKA result (*p*<0.05) at *RcH1.3* in the LC population indicates this locus may be experiencing balancing selection (selection for multiple alleles thus usually enhancing genetic diversity) (Kreitman, 2000; Nielsen, 2005). For *RcH1.1*, we found similar θw in the wild and LC population and slightly higher π in the LC population relative to the wild (**Figure 4**). As was the case for *RcH1.3*, significant evidence for departure from neutrality was detected using Tajima’s *D* (*D*=2.99) and the ML-HKA test (*p*<0.05, **Table 3**) in the LC population. Together this suggests that *RcH1.1* is probably experiencing balancing selection. The ratio of π_non-syn_/π_syn >_1 at *RcH1.1* and *RcH1.2b* in both wild and LC population (**Table 3**) suggest positive selection or the absence of removal of potentially deleterious alleles at these loci. We further analyzed the amino acid substitutions of the two *RcH1.1* and *RcH1.3* with balancing selection in the LC population, resulting in two non-synonymous SNPs (K169R and L234P) occurred in the *RcH1.1*, and only one non-synonymous SNP (D126E) occurred in the *RcH1.3*. All these varied amino acids were located at the C-terminal domain, but these variations did not cause the change of charge in CTD.

Whilst histone-related genes tend to be highly conserved (Okada et al., 2005), the H1 linker histones showed a similar amount of genetic variability as a typical subset of genes involving in fatty acids biosynthesis pathway within castor bean (**Supplementary Table S5**).

## Discussion

Although genome-scale data have been obtained for dozens of species, the genome-wide identification and characterization of genes encoding H1 linker histones is relatively limited. To ameliorate this, based on genome-wide characterization and variation analyses in castor bean and its relatives in the Euphorbiaceae, our study provides new information in understanding variation and biological significance of linker histones in plants.

Many studies have demonstrated that the conserved basic binding sites on the surface of the GH1 domain and the presence of a lysine-rich C-terminal tail with evenly distributed and strong positive charges are crucial structures for linker histones in binding DNA (Ramakrishnan et al., 1993; Goytisolo et al., 1996; Zhou et al., 1998; Lu and Hansen, 2004; Stasevich et al., 2010; Zhou et al., 2013; Zhou et al., 2015). Based on these features, we identified five linker histones from 13 GH1-containing proteins in castor bean. The Myb domain and AT-hook motifs are also able to recognize and bind specific DNA sites and functioning (Jerzmanowski et al., 2000; Marian et al., 2003), however whether these proteins containing GH1 domain with MYB (GH1-Myb protein) and AT-hook motifs (GH1-HMGA protein) have functions of linking DNA remain uncertain. Phylogenetic analysis of the H1s, GH1-Myb and GH1-HMGA proteins in castor bean demonstrates ancient and distinct differentiation of the three protein groups, which manifests that phylogeny-based identification of linker histone H1s as well the other two protein groups is effective and applicable.

Further on, phylogeny-based category and nomenclature of linker histone H1 variants has been suggested to be necessary and efficient due to their high variation (Talbert et al., 2012; Kotliński et al., 2017). Based on the phylogenetic analysis of amino acid sequences of all the GH1-containing proteins, we identified and characterized the H1 linker histones in castor bean and other five members in Euphorbiaceaees (**Supplementary Figure 3**). In particular, five distinct sub-clades were generated in H1 linker histones, which provided important evidence for naming as H1.0, H1.1, H1.2, H1.3, and H1.4, representing five H1 variants following the classification of H1s in *Arabidopsis*. The five putative H1s in castor bean were found in H1.1, H1.2, H1.3, and H1.4 sub-clades, which were named as RcH1.1, RcH1.2a, RcH1.2b, RcH1.3, and RcH1.4 correspondingly. Genes *RcH1.2a* and *RcH1.2b* encode identical proteins. Since the transcript of *RcH1.2b* was barely detected in any tissue, it is probably not functional. Inspection of **Supplementary Table S1** reveals that *RcH1.2b* lacks non-coding region, indicating the lack of promoter region, therefore this gene is likely a pseudogene. This is backed up by the nucleotide diversity analysis where a large number of non-synonymous variants were found, indicating its inability to remove deleterious mutations. Similarly, four H1 variants (four *H1* genes) were found in annual mercury, and five H1 variants in jatropha (six *H1* genes), cassava (six *H1* genes), tung tree (eight *H1* genes), and rubber tree (nine *H1* genes). Duplication of *H1* genes varies among these species as presented in the ML tree (**Figure 2**), with larger genome species harboring more *H1* duplicates (**Table 2**). Our nomenclature well presents the number of H1 variants and *H1* genes in species, and genes that encode identical proteins.

In the ML tree, the five H1 subclades (H1.0, H1.1, H1.2, H1.3, and H1.4) included three new sub-clades where *Arabidopsis* members were absent (**Figure 2**). The three known H1 homologs in *Arabidopsis* (AtH1.1, AtH1.2 and AtH1.3) were grouped into two sub-clades (H1.1 and H1.3), with *AtH1.1* and *AtH1.2* both being *H1.1* genes (**Figure 2**). Therefore, the nomenclature proposed by Talbert et al. (2012) and Kotliński et al. (2017) based on phylogeny of H1s from *Arabidopsis* may require updating to fully represent the diversity of *H1* genes across plant species more broadly. As linker histones are highly variable among more divergent species (Jerzmanowski et al., 2000; Talbert et al., 2012; Kotliński et al., 2017), nomenclature based on the phylogeny of H1s from several related species (such as within the same family) may be more efficient and applicable for classification of linker histones across broader sampling and other families in plant.

Since linker histones are required to organize and maintain genome stability within cells, the broad expression of *RcH1*s (except for the pseudogene *RcH1.2b*) among different tissues indicates their broad participation in development across various tissues in castor bean. Linker histones are involved in floral development in tobacco (Prymakowska-Bosak et al., 1999; Przewloka et al., 2002) and *Arabidopsis* (Wierzbicki and Jerzmanowski, 2005), hence the particularly high expression of *RcH1.2a* and *RcH1.4* in floral organs suggest their involvement in floral development in castor bean. Thus, we provide additional support for the suggestion that linker histones are essential for reproductive development for plants, which is likely due to their specific roles in accurate meiosis (Prymakowska-Bosak et al., 1999; Lu et al., 2009). The higher expression of *RcH1.1* and *RcH1.3* in embryo suggested their involvement in embryo development, and this function has also been reported in mouse and *Drosophila*, which may be related to their potential roles in epigenetic modifications (Fan et al., 2005; Lu et al., 2009).

H1.3 orthologs are stress-inducible, including under drought and low-light in *Arabidopsis* (Ascenzi and Gantt, 1997; Rutowicz et al., 2015), and drought in tomato (Scippa et al., 2000). Our study revealed that *RcH1.3* was salt stress inducible, but not cold, heat and drought inducible. The tobacco H1-C, an ortholog of AtH1.3, was also not induced by a long-term drought stress (Przewloka et al., 2002). It therefore appears that stress inducible H1 variants are not inducible by all stresses. AtH1.3 has been found to bind chromatin with considerably higher dynamics than the main H1 variants (AtH1.1 and AtH1.2) (Rutowicz et al., 2015), and this may be due to the replacement of the two putative basic binding sites (Lys17 and Lys55) in the GH1 domain to neutral Asn in H1.3 (**Supplementary Figure 5**) and the shorter CTD length in H1.3 (**Supplementary Figure 7**) (Rutowicz et al., 2015). Therefore, H1.3 may regulate plant stress response through changing configuration of chromatin thus promoting expression of stress related genes (Rutowicz et al., 2015). More research is needed to illustrate the exact mechanisms of H1.3 differential regulation in plant response to stresses.

Similar to animals, plant linker histones possess an evolutionarily conserved GH1 domain. The NTD and CTD of H1 linker histones are quite divergent between different variants in both amino acid sequence and length, but they are highly conserved between species for a given variant. As shown in the six Euphorbiaceae species and *Arabidopsis thaliana*, the lysine content in CTD of plant linker histones is ~30%, lower than that in animals, which can be over 40% (Lu and Hansen, 2004). Although numbers of *H1* genes vary across species, the number of linker histone variants tends to be relatively conserved in species with closer phylogenetic relations (such as in the same family).

At the population level, genetic diversity θw for the five *H1* genes ranged from 0.0006-0.0017, which was equivalent to θw of the nine genes involving in fatty acids biosynthesis pathway (including *FAD3*, *KAS II*, *DGAT1*, *Ole2*, *CPT*, *PAP*, *KAS I*, *GPDH*, and *LPA*; **Supplementary Table S5**), therefore the H1 linker histones are not any more or less variable than a typical subset of genes. The stress-inducible gene *RcH1.3* exhibited higher genetic diversity than the other genes (excluding the likely pseudogene *RcH1.2b*), especially in the domesticated lines, which appears to be a result of balancing selection. Since H1.3 involves in plant stress regulation, the balancing selection on this gene could result from different alleles being beneficial in different environments. A total of 95 SNPs were detected at *RcH1.3* from the 85 LC samples from 28 countries, with the latitude ranging from S 34.94° to N55.74° and longitude from W125.40°to E128.90°; whilst 78 SNPs were detected in the 106 wild samples from Kenya and Ethiopia (the two countries are the center of wild castor bean). Therefore, compared to the wild lines, the cultivated accessions are more widely spread in the world, thus facing a more heterogeneous environment. This might be the reason that *RcH1.3* exhibited higher genetic diversity in domesticated lines. Evidence of balancing selection also was detected at *RcH1.1*. Since the specific role of RcH1.1 is unclear at present, the biological relevance of variation at *RcH1.1* remains unknown. We observed higher expression of *RcH1.1* in the embryo which might suggest its roles in adaption to the environment in terms of inheritable character in embryo, although this remains untested. However, we did not find distinct evidence that the amino acid substitutions resulted in change of histone RcH1.1 and RcH1.3 as DNA linkers. The specific biological significance of amino acid substitutions within linker histones under selection remains unknown.

## Material and methods

### Identification of the GH1-containing proteins in castor bean genome

Hidden Markov Model (HMM) analysis was used to the search for GH1-containing proteins in castor bean. HMM profile of linker histones H1 and H5 (PF00538) from the Pfam protein family database were downloaded, and used as query to search the castor bean protein sequence data (http://oilplants.iflora.cn/Download/castor_download.html, Rc039) with a threshold of *p* < 0.01. To avoid missing probable members, amino acid sequences of H1.1, H1.2, and H1.3 from *Arabidopsis thaliana* listed in Kotliński et al. (2017) were downloaded from TAIR (http://www.arabidopsis.org/) and used as query sequences against castor bean protein database by running the ncbi-blast-2.12.0+ software (2.12.0+, https://ftp.ncbi.nlm.nih.gov/blast/executables/blast+/LATEST/) with an e-value < 0.001. All protein sequences which were returned were then checked for the presence of GH1 domains using SMART (http://smart.embl-heidelberg.de/) and Pfam (http://www.sanger.ac.uk/Software/Pfam/) databases. Domain architecture analysis was also carried out in SMART. The GH1-containing proteins in the five Euphorbiaceae species (*Manihot esculenta*, *Jatropha curcas*, *Hevea brasiliensis*, *Mercurialis annua*, and *Vernicia fordii*), were identified using the same approaches as for castor bean. Protein sequences of *Manihot esculenta* were downloaded from phytozome12: https://phytozome.jgi.doe.gov/pz/portal.html , *Jatropha curcas* downloaded from http://www.kazusa.or.jp/jatropha/ , *Hevea brasiliensis* downloaded from https://www.ncbi.nlm.nih.gov/genome/ (Assembly name ASM165405v1) , *Mercurialis annua* downloaded from https://osf.io/a9wjb/, and *Vernicia fordii* downloaded from https://ngdc.cncb.ac.cn/search/ (accession number GWHAAEU00000000). Again sequences of GH1-containing proteins in *Arabidopsis thaliana* were downloaded from TAIR using the protein ID provided in Kotliński et al. (2017). The molecular weight (MW) and theoretical isoelectric point (pI) of proteins were predicted using the ExPASy (https://web.expasy.org/protparam/).

### Model building of GH1 domain

To identify the secondary structure of GH1 domains, amino acid sequences of all the GH1 containing proteins in castor bean were submitted to SWISS-MODEL (https://swissmodel.expasy.org/) for homology model building and the structure of *Gallus gallus* GH5 (1hst.1.A) was chose as the template. Then, the 3D model of GH1 was visualized in PyMOL (v2.5.2) (Delano, 2002).

### Moving sum plot of net charge

Moving sum plot of net charges for both N- and C-terminal regions of all the GH1-containing proteins in castor bean was generated. The net charges were summed in a 20-aa sliding window along N- and C-terminal tails, starting from GH1 domain.

### Phylogenetic analysis of the GH1-containing proteins

Maximum likelihood (ML) phylogenetic trees based on the amino acid sequences of GH1 domains and whole proteins of GH1-containing proteins from six Euphorbiaceae species and *Arabidopsis thaliana* were constructed respectively. Sequence alignment was conducted in MAFFT (v7.490) (Katoh and Standley, 2013). Using the ModelFinder module in IQ-TREE (v1.6.12) (Nguyen et al., 2014), the best fitted model of amino acid substitutions according to the Bayesian information criterion (BIC) were determined (JTT+R4 for the whole protein alignment and JTTDCMut+G4 for the GH1 domain alignment). ML trees were constructed using IQ-TREE (version 1.6.12) (Nguyen et al., 2014). ML phylogenetic trees were constructed for the identified linker histones from castor bean and other five Euphorbiaceae species using the amino acid sequences of whole proteins with the JTT+R4 model of amino acid substitutions. To assess branch support, the IQ-TREE analyses used the ultrafast bootstrap approximation (UFboot) with 10000 replicates (Minh et al., 2013) and approximate likelihood ratio test with 1000 replicates.

### Gene expression analysis

To investigate the expression profile of *H1*s in castor bean across different tissues and stresses, TPM values in 11 tissues and in a subset of tissues exposed to stresses were acquired from our castor bean database (https://woodyoilplants.iflora.cn/). The 11 tissues include ovule, inflorescence, pollen, seedling, embryo (40 days after pollination), pericarp, endosperm (40 days after pollination), stem, developing seed (25 days after pollination), leaf and root. Four stresses include cold, heat, drought, and salt. Castor bean seeds were germinated at 28°C, and each germinated seed was sown into one pot and cultivated in a greenhouse at 25°C during the day and 22°C at night. For drought stress, two-week-old seedlings were not watered for one week and grown in the greenhouse. For heat and cold stress, 3-week-old seedlings were treated. For heat stress, the seedlings were kept at 45°C (with water to maintain humidity) for 12h at night. For cold stress, the seedlings were kept at 4°C for 12h at night. The control seedlings were continually grown in the greenhouse until they were harvested. After these treatments, seedlings of each treatment and control were harvested separately. For salt stress, 2-week-old seedlings were separated into two groups randomly and transported into a hydroponics system; after two weeks’ adaption to hydrophonics, the control group was continually supplied with full Hoagland’s solution, while salt treatment group was supplied by full Hoagland’s solution with 100 mM NaCl for 24h. Leaves and roots of the salt treatment and control groups were collected separately after salt treatment. After samples were collected, they were rapidly frozen in liquid nitrogen and then stored at -80°C for RNAseq analysis.

### Nucleotide diversity and selection analyses

The SNPs and indels of the *RcH1* genes were obtained from our re-sequencing dataset (Xu et al., 2021). DNA sequences of each gene of individual sample were obtained by replacing SNP sites in the reference genome with the alternative allele using SAMtools and BCFtools (Danecek et al., 2021). Multiple sequence alignments were then generated using MAFFT (v7.490) (Katoh and Standley, 2013). Estimates of nucleotide polymorphism, including number of segregating sites (*S*), θw (from the number of polymorphic segregating sites), π, and Tajima’s *D* were obtained using DNASP software (version 6.12.03) (Rozas et al., 2017). In the maximum-likelihood (ML) version of HKA test (Wright and Charlesworth, 2004), sequence diversity of each *H1* gene was compared to that of the five neutral genes in our previous studies (Xu et al., 2019). First, a strictly neutral model was run, followed by a selection model in which each gene was compared to the five neutral genes. These tests were carried out separately for the wild and cultivated datasets. Two times of the difference in log-likelihoods of the models was then used in a χ^2^ test with one degree of freedom for statistical significance test.

## Data availability statement

The data presented in the study are deposited in the NCBI repository, accession numbers PRJNA787114 and PRJNA872766.

## Author contributions

Conceived and designed the experiments: JG and AL. Analyzed the data: JG, PL, and AY. Wrote the paper: JG and AL. Revised the paper: MC and AL. All authors contributed to the article and approved the submitted version.

## Funding

This study was supported by Yunnan Fundamental Research Projects (202201AU070072, 202201AU070205, 202102AE090012), Foundation of Yunnan Agricultural Basic Research (202101BD070001-033, 202101BD070001-126), Foundation of Yunnan Province Education Department (2022J0503, 2021J0167) and The open project Key Laboratory for Forest Resources Conservation and Utilization in the Southwest Mountains of China (KLESWFU-202010, KLESWFU-202009).

## Conflict of interest

The authors declare that the research was conducted in the absence of any commercial or financial relationships that could be construed as a potential conflict of interest.

## Publisher’s note

All claims expressed in this article are solely those of the authors and do not necessarily represent those of their affiliated organizations, or those of the publisher, the editors and the reviewers. Any product that may be evaluated in this article, or claim that may be made by its manufacturer, is not guaranteed or endorsed by the publisher.
