# Supplementary material for: Genome-wide characterization and evolutionary analysis of linker histones in castor bean (Ricinus communis)
Source: Front Plant Sci. 2022 Oct 21;13:1014418. doi: 10.3389/fpls.2022.1014418 (PMC9635857; doi:10.3389/fpls.2022.1014418)
Supplement: Supplementary file 1 [file DataSheet_1.pdf]

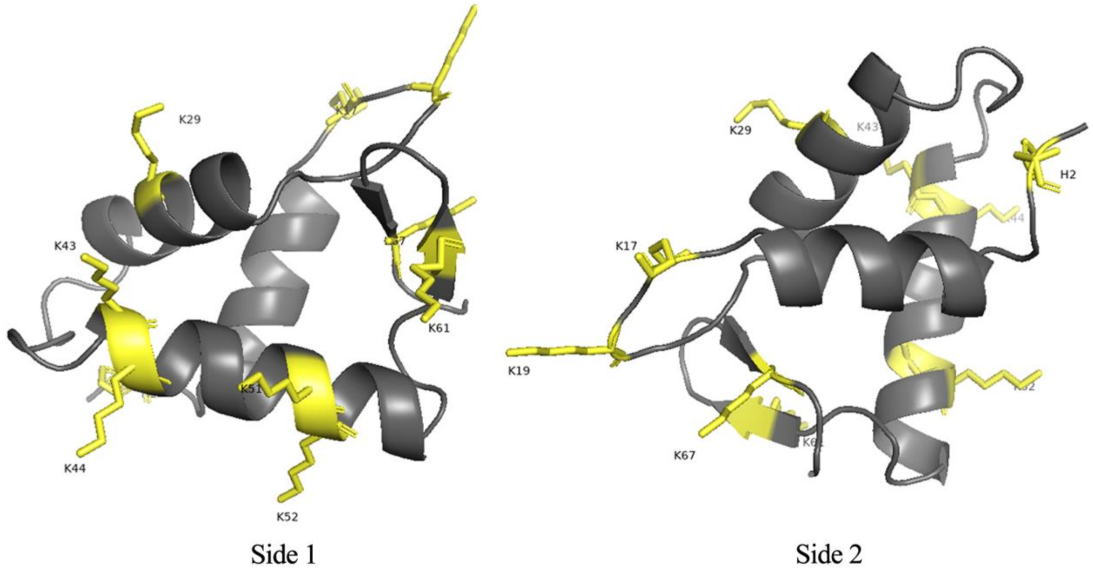

Supplementary Figure 1 3D model and binding residues (in yellow) in the GH1 domain of Rc01T000821.8.

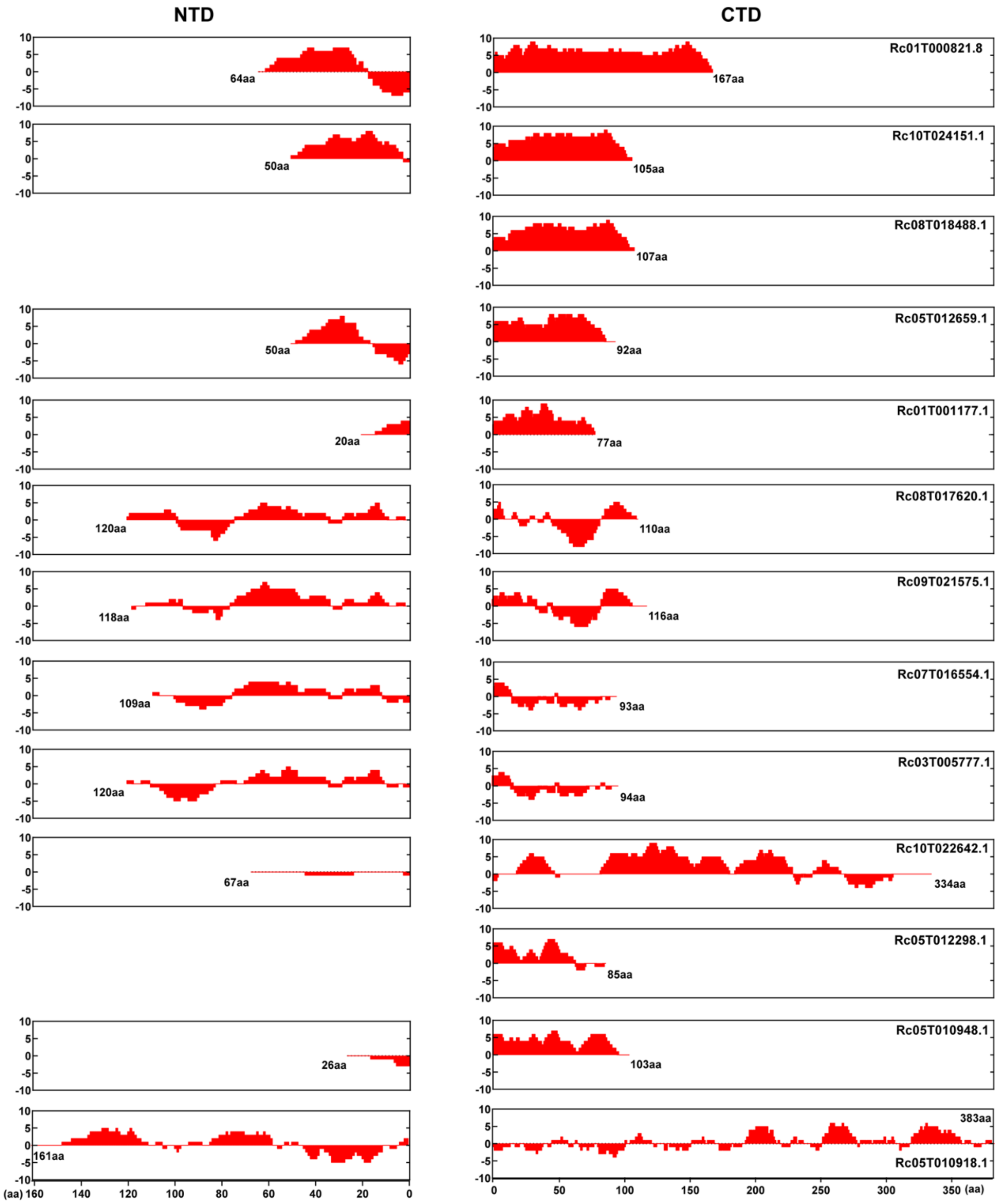

Supplementary Figure 2 Moving sum plot of net charge for N- and C-terminal domains (NTD and CTD) of all GH1-containing proteins in castor bean. The net charge (y-axis) is summed in a 20-aa sliding window along NTD and CTD.

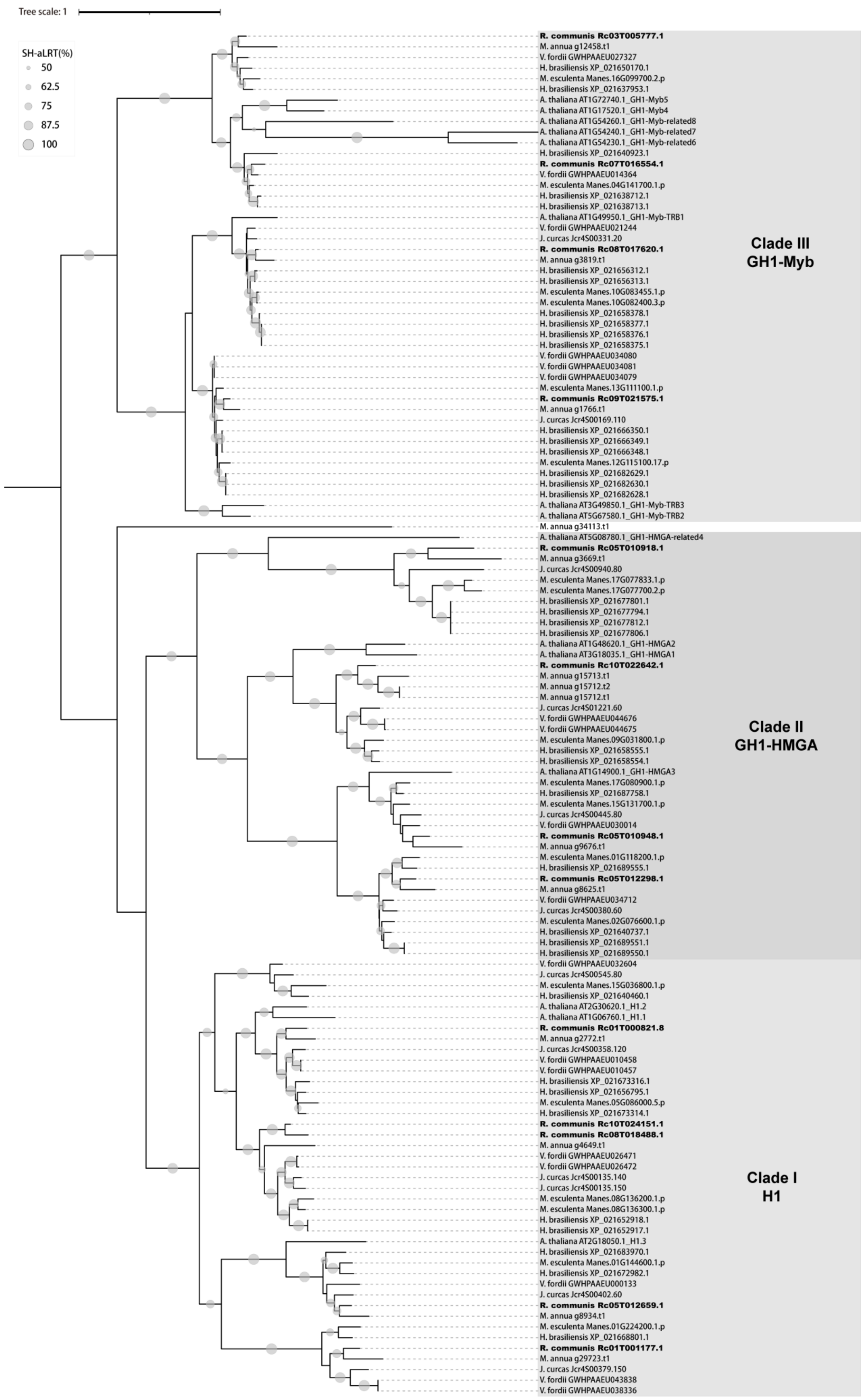

Tree scale: 1
SH-aLRT(%)
50
62.5
75
87.5
100
R. communis Rc03T005777.1
M. annua g12458.t1
V. fordii GWHPAAEU027327
H. brasiliensis XP_021650170.1
M. esculenta Manes.16G099700.2.p
H. brasiliensis XP_021637953.1
A. thaliana AT1G72740.1_GH1-Myb5
A. thaliana AT1G17520.1_GH1-Myb4
A. thaliana AT1G54260.1_GH1-Myb-related8
A. thaliana AT1G54240.1_GH1-Myb-related7
A. thaliana AT1G54230.1_GH1-Myb-related6
H. brasiliensis XP_021640923.1
R. communis Rc07T016554.1
V. fordii GWHPAAEU014364
M. esculenta Manes.04G141700.1.p
H. brasiliensis XP_021638712.1
H. brasiliensis XP_021638713.1
A. thaliana AT1G49950.1_GH1-Myb-TRB1
V. fordii GWHPAAEU021244
J. curcas Jcr4S00331.20
R. communis Rc08T017620.1
M. annua g3819.t1
H. brasiliensis XP_021656312.1
H. brasiliensis XP_021656313.1
M. esculenta Manes.10G083455.1.p
M. esculenta Manes.10G082400.3.p
H. brasiliensis XP_021658378.1
H. brasiliensis XP_021658377.1
H. brasiliensis XP_021658376.1
H. brasiliensis XP_021658375.1
V. fordii GWHPAAEU034080
V. fordii GWHPAAEU034081
V. fordii GWHPAAEU034079
M. esculenta Manes.13G111100.1.p
R. communis Rc09T021575.1
M. annua g1766.t1
J. curcas Jcr4S00169.110
H. brasiliensis XP_021666350.1
H. brasiliensis XP_021666349.1
H. brasiliensis XP_021666348.1
M. esculenta Manes.12G115100.17.p
H. brasiliensis XP_021682629.1
H. brasiliensis XP_021682630.1
H. brasiliensis XP_021682628.1
A. thaliana AT3G49850.1_GH1-Myb-TRB3
A. thaliana AT5G67580.1_GH1-Myb-TRB2
M. annua g34113.t1
A. thaliana AT5G08780.1_GH1-HMGA-related4
R. communis Rc05T010918.1
M. annua g3669.t1
J. curcas Jcr4S00940.80
M. esculenta Manes.17G077833.1.p
M. esculenta Manes.17G077700.2.p
H. brasiliensis XP_021677801.1
H. brasiliensis XP_021677794.1
H. brasiliensis XP_021677812.1
H. brasiliensis XP_021677806.1
A. thaliana AT1G48620.1_GH1-HMGA2
A. thaliana AT3G18035.1_GH1-HMGA1
R. communis Rc10T022642.1
M. annua g15713.t1
M. annua g15712.t2
M. annua g15712.t1
J. curcas Jcr4S01221.60
V. fordii GWHPAAEU044676
V. fordii GWHPAAEU044675
M. esculenta Manes.09G031800.1.p
H. brasiliensis XP_021658555.1
H. brasiliensis XP_021658554.1
A. thaliana AT1G14900.1_GH1-HMGA3
M. esculenta Manes.17G080900.1.p
H. brasiliensis XP_021687758.1
M. esculenta Manes.15G131700.1.p
J. curcas Jcr4S00445.80
V. fordii GWHPAAEU030014
R. communis Rc05T010948.1
M. annua g9676.t1
M. esculenta Manes.01G118200.1.p
H. brasiliensis XP_021689555.1
R. communis Rc05T012298.1
M. annua g8625.t1
V. fordii GWHPAAEU034712
J. curcas Jcr4S00380.60
M. esculenta Manes.02G076600.1.p
H. brasiliensis XP_021640737.1
H. brasiliensis XP_021689551.1
H. brasiliensis XP_021689550.1
V. fordii GWHPAAEU032604
J. curcas Jcr4S00545.80
M. esculenta Manes.15G036800.1.p
H. brasiliensis XP_021640460.1
A. thaliana AT2G30620.1_H1.2
A. thaliana AT1G06760.1_H1.1
R. communis Rc01T000821.8
M. annua g2772.t1
J. curcas Jcr4S00358.120
V. fordii GWHPAAEU010458
V. fordii GWHPAAEU010457
H. brasiliensis XP_021673316.1
H. brasiliensis XP_021656795.1
M. esculenta Manes.05G086000.5.p
H. brasiliensis XP_021673314.1
R. communis Rc10T024151.1
R. communis Rc08T018488.1
M. annua g4649.t1
V. fordii GWHPAAEU026471
V. fordii GWHPAAEU026472
J. curcas Jcr4S00135.140
J. curcas Jcr4S00135.150
M. esculenta Manes.08G136200.1.p
M. esculenta Manes.08G136300.1.p
H. brasiliensis XP_021652918.1
H. brasiliensis XP_021652917.1
A. thaliana AT2G18050.1_H1.3
H. brasiliensis XP_021683970.1
M. esculenta Manes.01G144600.1.p
H. brasiliensis XP_021672982.1
V. fordii GWHPAAEU000133
J. curcas Jcr4S00402.60
R. communis Rc05T012659.1
M. annua g8934.t1
M. esculenta Manes.01G224200.1.p
H. brasiliensis XP_021668801.1
R. communis Rc01T001177.1
M. annua g29723.t1
J. curcas Jcr4S00379.150
V. fordii GWHPAAEU043838
V. fordii GWHPAAEU038336
Clade III
GH1-Myb
Clade II
GH1-HMGA
Clade I
H1

Supplementary Figure 3 Maximum likelihood phylogenetic tree based on amino acid sequences of GH1-containing proteins in *Ricinus communis*, *Hevea brasiliensis*, *Jatropha curcas*, *Manihot esculenta*, *Mercurialis annua*, *Vernicia fordii* and *Arabidopsis thaliana*. The branches labeled by light grey circles indicate > 50% SH-like supports of approximate likelihood ratio test (SH-aLRT) and over 70% ultrafast bootstrap support.

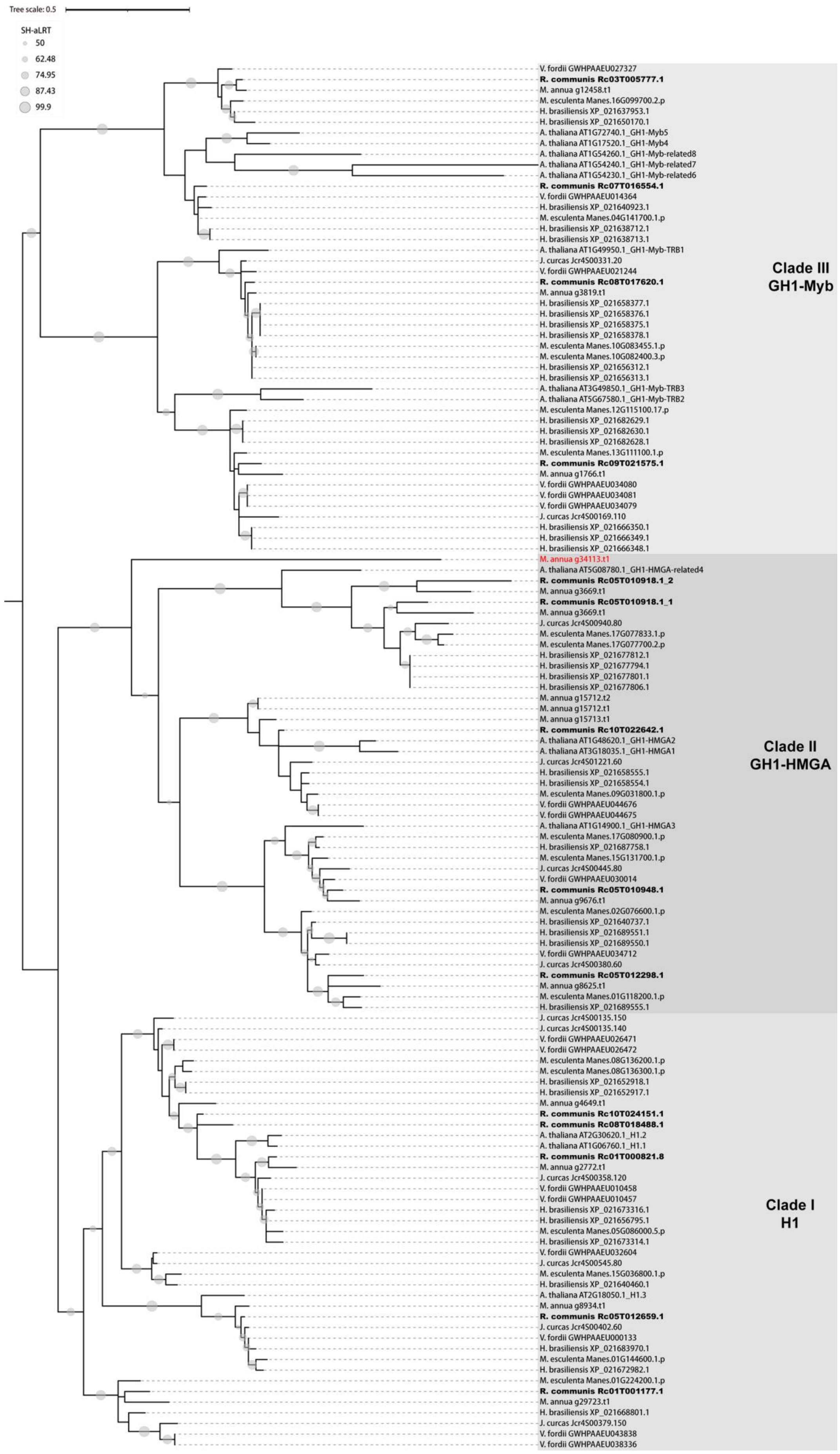

Tree scale: 0.5
SH-aLRT
50
62.48
74.95
87.43
99.9
V. fordii GWHPAAEU027327
R. communis Rc03T005777.1
M. annua g12458.t1
M. esculenta Manes.16G099700.2.p
H. brasiliensis XP_021637953.1
H. brasiliensis XP_021650170.1
A. thaliana AT1G72740.1_GH1-Myb5
A. thaliana AT1G17520.1_GH1-Myb4
A. thaliana AT1G54260.1_GH1-Myb-related8
A. thaliana AT1G54240.1_GH1-Myb-related7
A. thaliana AT1G54230.1_GH1-Myb-related6
R. communis Rc07T016554.1
V. fordii GWHPAAEU014364
H. brasiliensis XP_021640923.1
M. esculenta Manes.04G141700.1.p
H. brasiliensis XP_021638712.1
H. brasiliensis XP_021638713.1
A. thaliana AT1G49950.1_GH1-Myb-TRB1
J. curcas Jcr4S00331.20
V. fordii GWHPAAEU021244
R. communis Rc08T017620.1
M. annua g3819.t1
H. brasiliensis XP_021658377.1
H. brasiliensis XP_021658376.1
H. brasiliensis XP_021658375.1
H. brasiliensis XP_021658378.1
M. esculenta Manes.10G083455.1.p
M. esculenta Manes.10G082400.3.p
H. brasiliensis XP_021656312.1
H. brasiliensis XP_021656313.1
A. thaliana AT3G49850.1_GH1-Myb-TRB3
A. thaliana AT5G67580.1_GH1-Myb-TRB2
M. esculenta Manes.12G115100.17.p
H. brasiliensis XP_021682629.1
H. brasiliensis XP_021682630.1
H. brasiliensis XP_021682628.1
M. esculenta Manes.13G111100.1.p
R. communis Rc09T021575.1
M. annua g1766.t1
V. fordii GWHPAAEU034080
V. fordii GWHPAAEU034081
V. fordii GWHPAAEU034079
J. curcas Jcr4S00169.110
H. brasiliensis XP_021666350.1
H. brasiliensis XP_021666349.1
H. brasiliensis XP_021666348.1
M. annua g34113.t1
A. thaliana AT5G08780.1_GH1-HMGA-related4
R. communis Rc05T010918.1_2
M. annua g3669.t1
R. communis Rc05T010918.1_1
M. annua g3669.t1
J. curcas Jcr4S00940.80
M. esculenta Manes.17G077833.1.p
M. esculenta Manes.17G077700.2.p
H. brasiliensis XP_021677812.1
H. brasiliensis XP_021677794.1
H. brasiliensis XP_021677801.1
H. brasiliensis XP_021677806.1
M. annua g15712.t2
M. annua g15712.t1
M. annua g15713.t1
R. communis Rc10T022642.1
A. thaliana AT1G48620.1_GH1-HMGA2
A. thaliana AT3G18035.1_GH1-HMGA1
J. curcas Jcr4S01221.60
H. brasiliensis XP_021658555.1
H. brasiliensis XP_021658554.1
M. esculenta Manes.09G031800.1.p
V. fordii GWHPAAEU044676
V. fordii GWHPAAEU044675
A. thaliana AT1G14900.1_GH1-HMGA3
M. esculenta Manes.17G080900.1.p
H. brasiliensis XP_021687758.1
M. esculenta Manes.15G131700.1.p
J. curcas Jcr4S00445.80
V. fordii GWHPAAEU030014
R. communis Rc05T010948.1
M. annua g9676.t1
M. esculenta Manes.02G076600.1.p
H. brasiliensis XP_021640737.1
H. brasiliensis XP_021689551.1
H. brasiliensis XP_021689550.1
V. fordii GWHPAAEU034712
J. curcas Jcr4S00380.60
R. communis Rc05T012298.1
M. annua g8625.t1
M. esculenta Manes.01G118200.1.p
H. brasiliensis XP_021689555.1
J. curcas Jcr4S00135.150
J. curcas Jcr4S00135.140
V. fordii GWHPAAEU026471
V. fordii GWHPAAEU026472
M. esculenta Manes.08G136200.1.p
M. esculenta Manes.08G136300.1.p
H. brasiliensis XP_021652918.1
H. brasiliensis XP_021652917.1
M. annua g4649.t1
R. communis Rc10T024151.1
R. communis Rc08T018488.1
A. thaliana AT2G30620.1_H1.2
A. thaliana AT1G06760.1_H1.1
R. communis Rc01T000821.8
M. annua g2772.t1
J. curcas Jcr4S00358.120
V. fordii GWHPAAEU010458
V. fordii GWHPAAEU010457
H. brasiliensis XP_021673316.1
H. brasiliensis XP_021656795.1
M. esculenta Manes.05G086000.5.p
H. brasiliensis XP_021673314.1
V. fordii GWHPAAEU032604
J. curcas Jcr4S00545.80
M. esculenta Manes.15G036800.1.p
H. brasiliensis XP_021640460.1
A. thaliana AT2G18050.1_H1.3
M. annua g8934.t1
R. communis Rc05T012659.1
J. curcas Jcr4S00402.60
V. fordii GWHPAAEU000133
H. brasiliensis XP_021683970.1
M. esculenta Manes.01G144600.1.p
H. brasiliensis XP_021672982.1
M. esculenta Manes.01G224200.1.p
R. communis Rc01T001177.1
M. annua g29723.t1
H. brasiliensis XP_021668801.1
J. curcas Jcr4S00379.150
V. fordii GWHPAAEU043838
V. fordii GWHPAAEU038336
Clade III
GH1-Myb
Clade II
GH1-HMGA
Clade I
H1

Supplementary Figure 4 Maximum likelihood phylogenetic tree based on amino acid sequences of the GH1 domain in GH1-containing proteins from *Ricinus communis*, *Hevea brasiliensis*, *Jatropha curcas*, *Manihot esculenta*, *Mercurialis annua*, *Vernicia fordii*, and *Arabidopsis thaliana* based on the JTTDCMut+G4 model of amino acid substitutions. The branches labeled by light grey circles indicate over 50% SH-like supports of approximate likelihood ratio test (SH-aLRT) and over 70% ultrafast bootstrap support.

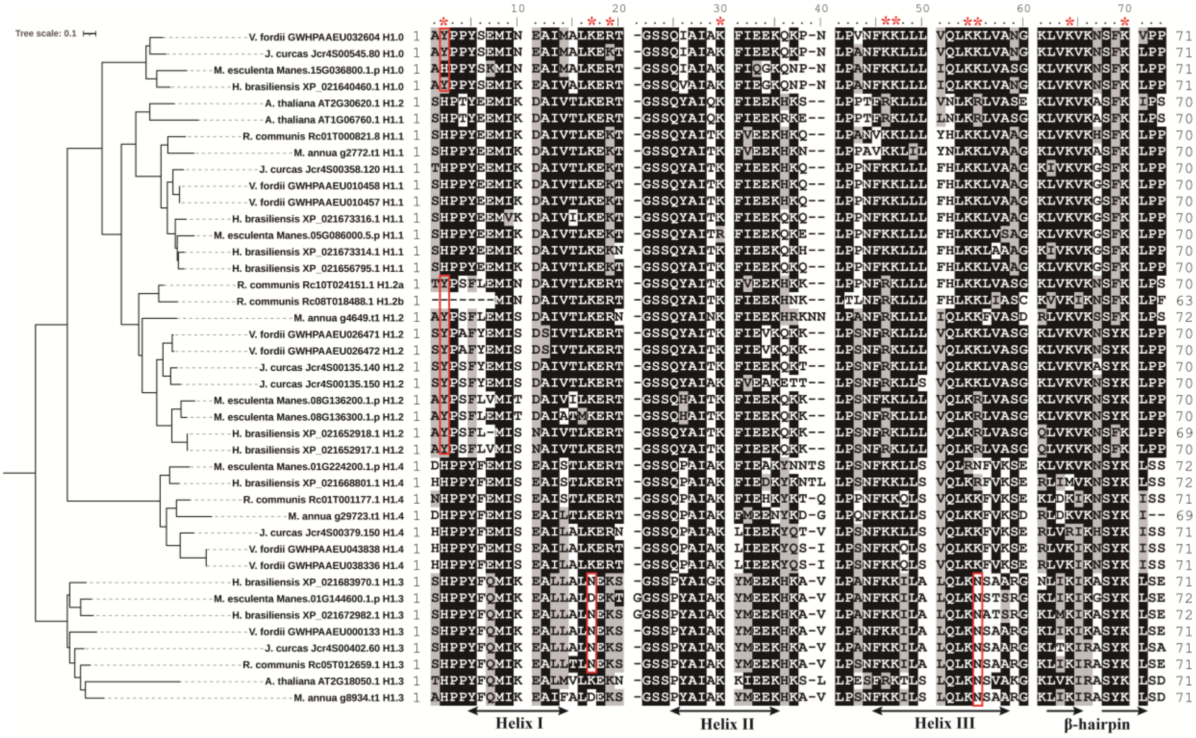

Supplementary Figure 5 Comparisons of amino acid sequence of the GH1 domain between H1s in different species. The sites with asterisks above are the putative conserved basic residue sites of the GH1 in H1s. Red boxes indicate potential binding sites that have diverged between the five subgroups (H1.0-H1.4).

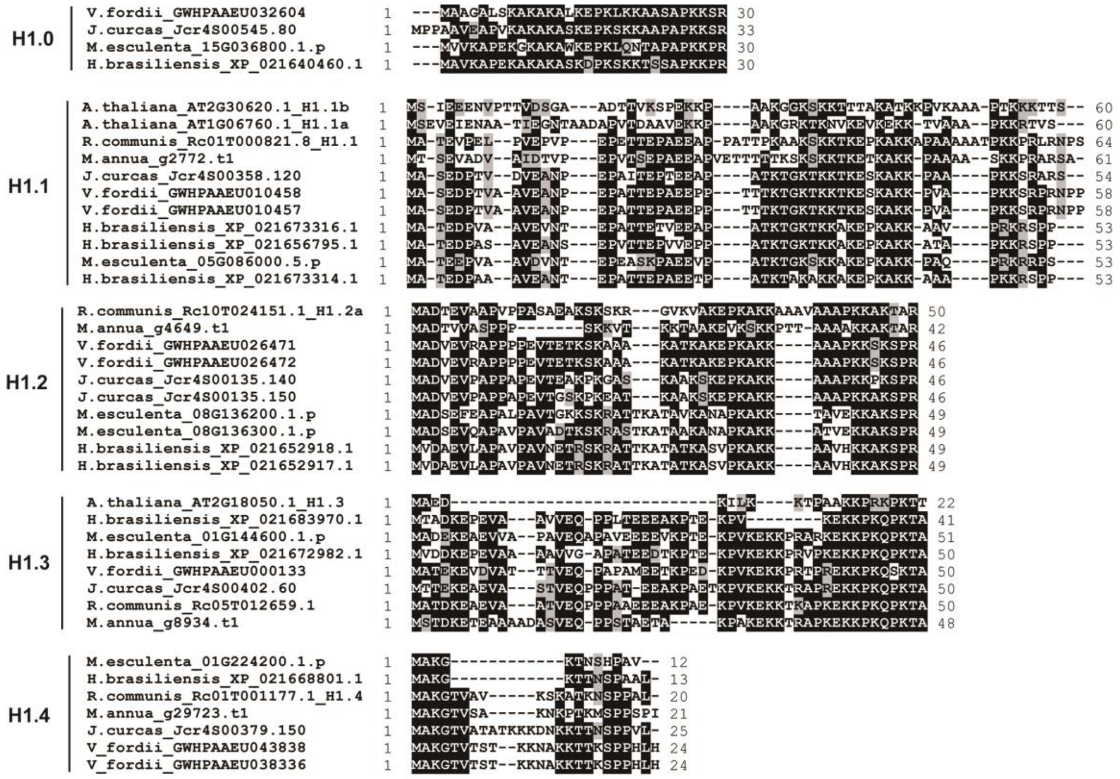

Supplementary Figure 6 Sequence comparisons of NTD in the H1s from the seven species.

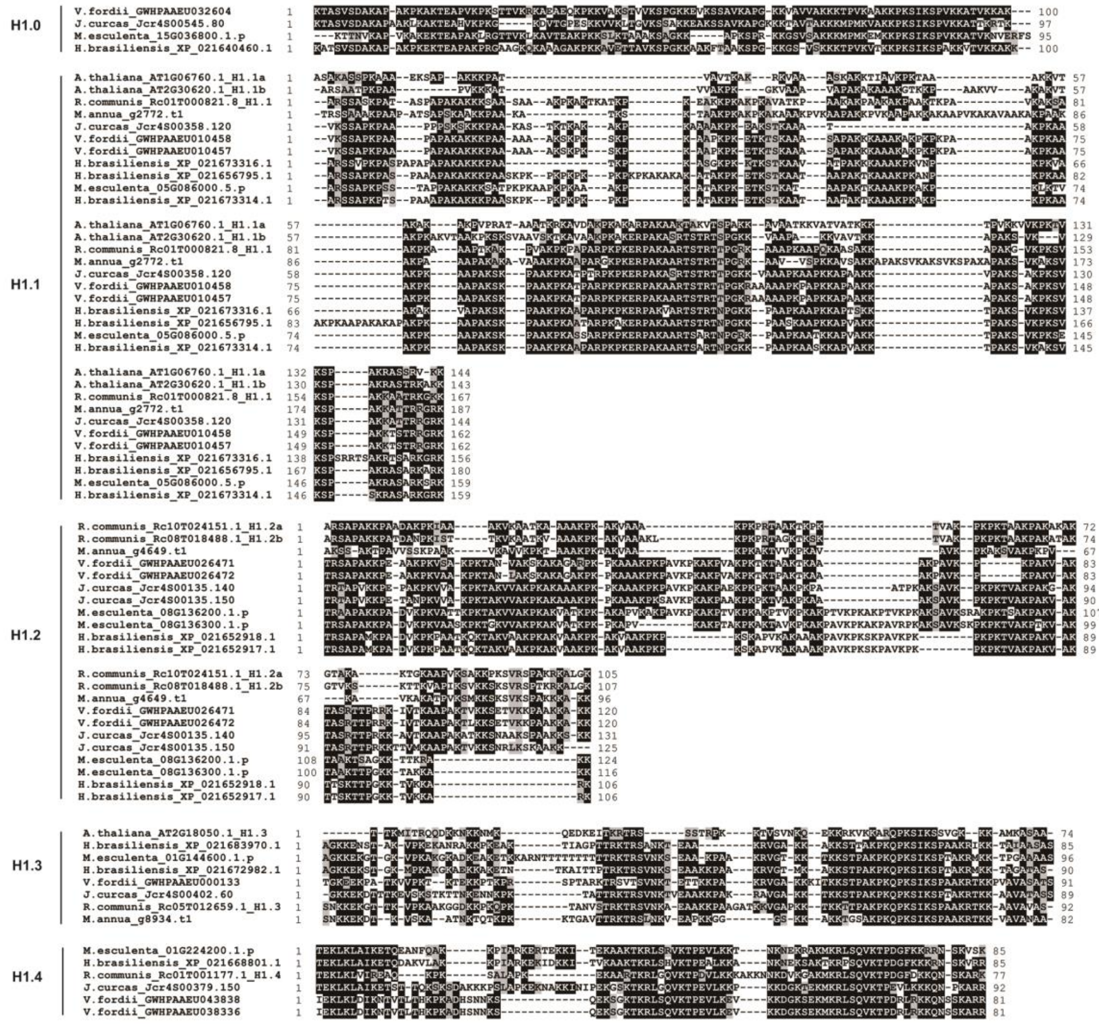

Supplementary Figure 7 Sequence comparisons of CTD in the H1s from the seven species.
